# Circadian influences on myocardial infarction

**DOI:** 10.3389/fphys.2014.00422

**Published:** 2014-10-30

**Authors:** Jitka A. I. Virag, Robert M. Lust

**Affiliations:** Department of Physiology, Brody School of Medicine, East Carolina UniversityGreenville, NC, USA

**Keywords:** myocardial infarction, circadian rhythm, acute ischemia, remodeling, cardioprotection

## Abstract

Components of circadian rhythm maintenance, or “clock genes,” are endogenous entrainable oscillations of about 24 h that regulate biological processes and are found in the suprachaismatic nucleus (SCN) and many peripheral tissues, including the heart. They are influenced by external cues, or Zeitgebers, such as light and heat, and can influence such diverse phenomena as cytokine expression immune cells, metabolic activity of cardiac myocytes, and vasodilator regulation by vascular endothelial cells. While it is known that the central master clock in the SCN synchronizes peripheral physiologic rhythms, the mechanisms by which the information is transmitted are complex and may include hormonal, metabolic, and neuronal inputs. Whether circadian patterns are causally related to the observed periodicity of events, or whether they are simply epi-phenomena is not well established, but a few studies suggest that the circadian effects likely are real in their impact on myocardial infarct incidence. Cycle disturbances may be harbingers of predisposition and subsequent response to acute and chronic cardiac injury, and identifying the complex interactions of circadian rhythms and myocardial infarction may provide insights into possible preventative and therapeutic strategies for susceptible populations.

## Clinical associations for circadian rhythms in myocardial infarction

The ability to properly synchronize with the external environment is an essential element of animal survival. The predation rate of free-living chipmunks increased over time in suprachiasmatic nucleus (SCN)-lesioned animals due to their reduced burrowing during the nighttime (Decoursey et al., 2000). On the other hand, rats kept in total darkness during the first 48 h following brain injury exhibited improved recovery (Corwin and Vargo, 1993; Vargo et al., 1998, 1999). In humans, disruptions in rhythms are associated with reduced survival of patients with metastatic cancers (Mormont et al., 2000; Sephton et al., 2000) and alterations in expression of clock genes have been shown to be responsible for unregulated cell growth (Granda et al., 2005; Chu et al., 2008; Clairambault, 2008; Yang et al., 2008).

The significance of circadian rhythms to myocardial infarction has been identified in several studies in which onset of non-Q-wave angina, unstable angina, myocardial infarctions, and sudden cardiac death all show marked elevations in occurrence between the hours of 0600 and 1200 (Muller, 1999a,b; Willich et al., 2004; Mosendane and Raal, 2008). Ethnic genetic, medications, co-morbidities, lifestyle, cultural, and/or social factors may shift the time of highest incidence (Kanth et al., 2013). Lopez et al. (2005) and Savopoulos et al. (2006) each reported a significant shift to the 1800–2400 h window for increased incidence of sudden death in Mediterranean populations. The incidence of ischemic stroke, has been reported to follow the same patterns (Gupta and Shetty, 2005), with highest incidence in the 0600–1200 window and diurnal variation in ventricular arrhythmias have recently been demonstrated to be related to abnormal repolarization (Jeyaraj et al., 2012). The time of day when an event occurs also may influence the severity of the outcome (Mukamal et al., 2000) and also may extend to the timing of salvaging intervention. The time of day when mechanical reperfusion by percutaneous transluminal coronary angioplasty (PTCA) is performed influences subsequent 1 year mortality rates, with patients treated between 0400 and 0800 having worse outcomes than all others, even after correcting for confounding variables (De Luca et al., 2005).

Blood pressure normally follows a diurnal pattern, with pressures lowest at night, and rising to a peak in the hours 0600–1000. Several ambulatory blood pressure studies have identified that failure to decrease blood pressure at night, so called “non-dipping,” is associated with increased cardiovascular risk (Morgan, 2002; Lee et al., 2005; Izzedine et al., 2006), and optimal efficacy in relation to time of day is a major factor considered in anti-hypertensive drug development (Lemmer, 2006). More recently, it has been shown that Per2 mutation diminishes blood pressure and heart rate dipping (Vukolic et al., 2010). Many intrinsic vasoactive and cardioactive substances, such as angiotensin II, melatonin, plasminogen activator inhibitor 1 (*pai-1*), glucocorticoids, epinephrine, norepinephrine, and nitric oxide follow distinct circadian patterns (Ding et al., 1994; Balsalobre et al., 2000; McNamara et al., 2001; Nonaka et al., 2001; Naito et al., 2003; Tsuchiya et al., 2005; Tsujino et al., 2005; Vaughan, 2005; Kanth et al., 2013). To the extent that diabetes and obesity are contributing factors to cardiovascular risk, recent reviews associating circadian genes with metabolic dysfunction in peripheral tissues are noteworthy. And whether these many circadian patterns are causally related to the observed periodicity of events, or whether they are simply epiphenomena is not well established, but a few studies suggest that the circadian effects likely are real in their impact on myocardial infarction incidence (Bray and Young, 2007; Green et al., 2008; Prasai et al., 2008; Ruger and Scheer, 2009; Durgan and Young, 2010; Maury et al., 2010; Bailey et al., 2014; Nedeltcheva and Scheer, 2014). Chronic instability in circadian rhythm in shift worker studies was recognized more than 20 years ago to produce increased risk of cardiovascular morbidity and mortality (Knutsson et al., 1986). More recently, Fujino et al. (2006) reported that the relative risk for ischemic heart disease in Japanese factory workers on rotating shifts doubles compared to that of workers with either fixed daytime or fixed night-time schedules. Kawachi et al. reported similar findings in a study of U.S. nurses, with the multivariate risk increasing commensurate with the duration of rotating shift work (Kawachi et al., 1995). Notably, in hamsters with a mutation producing a 22 h circadian cycle, premature cardiac mortality was noted, and corrected when either the animals were converted to a 22 h L/D cycle matching the phenotype, or the SCN was ablated, abolishing the dys-synchrony (Martino et al., 2008). Further, the same group also demonstrated that when mice were maintained in a disrupted light/dark (L/D) rhythm (10/10 vs. normal 12/12), the hypertrophy response to aortic banding was badly impaired (Martino et al., 2007).

These findings are consistent with the premise that synchronization between the central and peripheral clocks is a continuous, ongoing process, and that the pressure to maintain this synchronization involves signaling mechanisms that are energetically demanding for the peripheral target tissues. When coupled with an underlying pathophysiology that generates a vulnerable substrate, such as coronary artery disease or pressure overload, the pressure to normalize desynchronized rhythms increases the progression of injury.

## Circadian rhythm biology

Circadian rhythms are daily variations of physiological functions that are found in every living organism. These daily rhythms are generated through the integration of oscillatory expression of multiple circadian clock genes (Harmer et al., 2001; Dvornyk et al., 2003; Merrow et al., 2005; Granados-Fuentes and Herzog, 2013). In mammals, the circadian rhythms are regulated by the SCN of the hypothalamus. Neurons in the SCN generate self-sustained daily oscillations of gene expression and electrical activity with a frequency of close to 24 h (Herzog and Schwartz, 2002). The SCN keeps the circadian rhythms of different peripheral organs synchronized to each other as well as to the environmental light-dark cycle (Dardente and Cermakian, 2005). Although every mammalian cell is believed to express the circadian clock genes, it has been shown that some cells outside the SCN are not able to maintain self-sustained circadian oscillation in the absence of the SCN (Fukuhara and Tosini, 2003).

Major progress has been made in identifying genes that regulate the circadian clock (Hardin and Glossop, 1999; Reppert and Weaver, 2002; Hardin, 2006; Hardin and Panda, 2013). The first circadian clock gene was identified in drosophila, named *Period* (*per*) (Kyriacou and Hall, 1980). The first mammalian counterpart of the *Period* gene was discovered by Sun et al. (1997). Molecular components of the circadian clock have been identified in mammals and interact to contribute to the entrainment and pace making of the circadian rhythm. These genes include a clock gene, a gene encoding brain-muscle Arnt-like protein 1 (bmal1) (Honma et al., 1998), three period genes (per1, per2, and per3) (Sun et al., 1997; Zheng et al., 1999; Shearman et al., 2000), and two cryptochrome genes (cry1 and cry2) (Thresher et al., 1998; Hardin and Glossop, 1999; Sancar, 2000). In addition, there is evidence that the NPAS2 gene, which encodes a functional analog of the clock, may play a role in the functioning of the circadian clock in the brain as well as peripheral organs (Reick et al., 2001; Hardin, 2006).

These clock genes participate in the pacemaking and phase regulation of the circadian rhythm. In the mammalian cells, CLOCK/BMAL1 form heterodimers that are positive regulators which activate the transcription of *per1, per2*, and *per3* through a transcription factor binding site, located in the promoter region of the *per* genes. *Per1, per2*, and *per3* mRNA are transcribed and move to the cytoplasm, where they are translated into PER1, PER2, and PER3 proteins. As the levels of PER1 and PER2 proteins increase and begin to form PER1/PER2 heterodimers, the protein complex enters the nuclei. However, nuclear translocation of the PER1/PER2 heterodimers is tightly regulated. Cryptochrome proteins, CRY1 and CRY2, negatively regulate clock feedback while PER3 enhances translocation of PER1 and PER2 to the nucleus (Sangoram et al., 1998; Glossop et al., 1999; Kume et al., 1999; Shearman et al., 2000; Yagita et al., 2000; Lee et al., 2001). *Doubletime* (or *casein kinase Ie* in mouse), can bind and phosphorylate PER1 and PER2, thereby masking PER1's nucleus localization signaling, promoting its retention in the cytoplasm and thereby its degradation (Glossop et al., 1999; Xu et al., 2007). In addition, CRY1/CRY2 act as light-independent inhibitors of CLOCK/BMAL1, thereby suppressing the *per* genes transcriptions (Sancar, 2000). This auto-regulatory feedback mechanism of clock gene expression serves as the basis for the temporal oscillation of the circadian clock (Hardin, 2006; Xu et al., 2007). REV-ERBA forms an accessory loop which periodically represses transcription of BMAL1, serving to stabilize the oscillator (Guillaumond et al., 2005; Reddy and Maywood, 2007). Although every mammalian cell is believed to express the circadian clock genes, it was held that cells outside the SCN could not maintain self-sustained circadian oscillation in the absence of the SCN (Fukuhara and Tosini, 2003). However, parabiosis studies and isolated cell culture studies clearly indicate that individual tissues can produce sustained intrinsic circadian rhythms independent of the SCN (Nagoshi et al., 2004, 2005; Davidson et al., 2005; Durgan et al., 2005; Guo et al., 2005). However, signals from the SCN or an environmental trigger are required to keep the individual cell rhythms in synchrony with each other. The mechanism of communication between the SCN and the bone marrow which produces regenerative cell populations that may assist in wound healing, the physiologic implications, and means to exploit circadian rhythms for clinical purposes are currently being investigated (Bourin et al., 2002; Tsinkalovsky et al., 2005, 2007; Scadden, 2008). There have been shown to be diurnal variations in granulocyte-colony stimulating factor (G-CSF) that alter the levels of circulating stem cells (Jilma et al., 1999). Mendez-Ferrer et al. (2008) showed that the circadian clock regulates bone marrow-derived SDF-1α via neuronal β_3_-adrenergic receptors.

It is clear that there is increased understanding of the basic nature by which a rhythmic gene expression can be produced. It is also clear that, other than output from a central clock, the manner by which a given peripheral clock is synchronized with another, or the extent to which a peripheral clock can establish feedback to the SCN that it is, in fact, synchronized is not known. At present, the literature does support the premise that pathophysiologic conditions can both cause uncoupling of peripheral oscillators and be caused by uncoupling of peripheral clocks (Muller, 1999a,b; Young et al., 2001a,b; Willich et al., 2004; Lee et al., 2005; Fujino et al., 2006; Maywood et al., 2010; Tsimakouridze et al., 2012; Reddy and Rey, 2014; Robinson and Reddy, 2014; Wu and Reddy, 2014).

## Circadian effects and acute ischemia/reperfusion injury

Ischemia/reperfusion (I/R) injury occurs when an artery that supplies the ventricular tissue becomes obstructed and limits blood flow, and thus oxygen and nutrients as well as waste removal. If this occlusion persists, the damage becomes irreversible. If flow can be reinstated (reperfusion), tissue can be salvaged but there is ancillary oxidative stress (Anaya-Prado et al., 2002; Kaminski et al., 2002; Hamacher-Brady et al., 2006). I/R injury is a complex process involving vascular and endothelial dysfunction, metabolic and mitochondrial dysfunction, necrosis, apoptosis, and functional deficits, even in tissue without permanent cellular injury (Jordan et al., 1999; Giordano, 2005; Seal and Gewertz, 2005; Cohen et al., 2006). A common feature of I/R injury is an inflammatory reaction, with infiltration of polymorphonuclear leukocytes (PMN), predominantly neutrophils which have been causally linked to myocardial damage during reperfusion injury (Jordan et al., 1999). Release of elastases, lipases, and proteases and peroxidases from azurophilic granules of neutrophils as well as reactive oxygen species (ROS) generated by NADPH oxidase in neutrophil membranes can result in direct myocyte damage (Rossi, 1986; Frangogiannis et al., 1996; Takayama et al., 2004). The interaction between neutrophils and endothelial cells is mediated by interactions at the vascular interface of different cell adhesion molecules including selectins, β_2_-integrins, and the immunoglobulin superfamily (Jones et al., 1999). Hypoxia and cytokines released from ischemic myocardium result in upregulation of these adhesion markers on endothelial surfaces as well as on the surface of neutrophils, thereby facilitating neutrophil recruitment to the tissue during reperfusion (Frangogiannis et al., 1996).

Inflammation plays a key role in the development and progression of myocardial infarction and neutrophil infiltration is a defining feature of ischemic cardiac diseases (Jordan et al., 1999). Reperfusion injury following revascularization procedures is a major cause of myocardial tissue damage, and, accordingly, reperfusion elevates proinflammatory cytokines and infiltration of neutrophils into the tissue (Welbourn et al., 1991; Frangogiannis et al., 1996).IL-1β and TNF-α have direct effects on cardiomyocytes (Suzuki et al., 2001; Chandrasekar et al., 2003), and TNF-α and IL-6 prime inflammatory cells, increasing their response to soluble proinflammatory mediators such as *N*-formyl peptides, C5a, and platelet-activating factors (Jordan et al., 1999). Infiltrated neutrophils cause tissue destruction by release of elastases, proteases, and superoxide radicals (Rossi, 1986; Rotrosen, 1992; Jordan et al., 1999). Many facets of immune function demonstrate diurnal patterns (Curtis et al., 2014).

Oxidant stress is a finely modulated event in normal physiology. The most common sources of routine oxidant burdens result from normal metabolic activity and mitochondrial electron transfer to molecular oxygen in the mitochondria at complexes I and III of the electron transport chain, producing superoxide and other ROS. Normally, excess ROS is scavenged and neutralized by the actions of enzymes like superoxide dismutase (SOD) and glutathione peroxidase (Taniyama and Griendling, 2003). Melatonin, a hormone with well-established circadian characteristics, has been reported to be a strong regulator of nitric oxide synthase (NOS) (Aydogan et al., 2006). Attenuated increases in night-time melatonin levels in hospitalized patients following angioplasty were correlated positively with increases in C-reactive peptide (CRP), an acute phase inflammatory marker, and were predictive of increased risk of event in the next 6 months (Dominguez-Rodriguez et al., 2006a,b). These findings, suggesting that melatonin is cardioprotective, and are consistent with reports that melatonin is an anti-oxidant, and stimulates anti-oxidant gene production (Hardeland et al., 2003). In isolated rat hearts, melatonin reduced necrosis and infarct size by inhibiting the mitochondrial permeability transition pore (MPTP) opening which reduced NAD+ and cytochrome c release (Petrosillo et al., 2009). However, Genade et al. reported that the addition of melatonin to isolated, perfused rat hearts abolished the cardioprotective effects induced by ischemic pre-conditioning (IPC) (Genade et al., 2006). The effect was attributed to melatonin's free radical scavenging ability, diminishing the level of ROS below those necessary to trigger the cardioprotective response. Nitric oxide, when produced in normal amounts, is an important signaling molecule, but when present in excess amounts may contribute to protein nitrosylation and ROS generation. In vascular disease, uncoupled activity of NOS, or production of ROS in excess of endogenous antioxidant capacity, leads to oxidative stress which turns into an abnormal vascular response (Cohen, 1995; Taniyama and Griendling, 2003; Lyle and Griendling, 2006). In I/R, a cascade of events occurs that prevents proper NOS-protein interactions, altering NOS function and NADPH oxidase activity, thereby altering myocyte function. However, two recent reviews highlight the fact that there remains significant disagreement regarding whether and how NO and isoforms of NOS contribute to, or inhibit cardioprotection, particularly in the setting of IPC (Cohen et al., 2006; Jones and Bolli, 2006). Lapenna et al. reported that glutathione peroxidase activity displayed circadian rhythm in the heart, which corresponded with sensitivity to H_2_O_2_-induced oxidative myocardial damage (Lapenna et al., 1992).

Circadian control of homeostatic functions is controlled by environmental and endogenous influences. Circadian genes have the capacity to regulate systems that modulate oxidant burdens, and therefore may potentially contribute significantly to the outcome of I/R or IPC-induced cardioprotection.

A central element of ischemia is hypoxia. The principal oxygen sensing molecule within the myocardium is hypoxia inducible factor 1 alpha (*HIF-1α*). *HIF-1α* is a basic helix-loop-helix PAS domain transcription factor. It is present as a cytosolic protein that is constitutively degraded, but under hypoxic conditions is phosphorylated, which stabilizes it, enabling nuclear translocation. In the nucleus *HIF-1α* forms heterodimers with the aryl hydrocarbon nuclear translocase (ARNT), or *HIF-1β*, and binds to hypoxia response elements in the promoter region of a wide range of genes, including those regulating angiogenesis vascular endothelial growth factor (VEGF), metabolism (especially genes coding for glycolytic enzymes), apoptosis, and vasomotor reactivity (NOS) (Semenza, 2001; Yamakawa et al., 2003; Giordano, 2005). Of particular note is that there is thought to be considerable homology in the binding characteristics of *Per2* and *HIF-1α*, such that cross talk has been reported between the circadian gene CLOCK, and *HIF-1α* in the regulation of vasopressin gene (Ghorbel et al., 2003). In addition, Koyonagi et al. reported that Per2 expression attenuated hypoxic induction of VEGF expression in a dose dependent manner (Koyanagi et al., 2003). The presumed mechanism was that *Per2* bound to *HIF-1α*, thus blocking nuclear translocation and preventing *HIF-1α* induced transcription of VEGF. Constitutively stable hybrids of *HIF-1α* were cardioprotective in cultured rat cardiomyocyctes exposed to simulated I/R injury (Date et al., 2005) and led to increases in several genes also induced by IPC, including HSP-70. However, HIF-1α also is known to be overexpressed in heart failure, which could represent a failure of a sustained attempt to compensate, or it could be that long-term expression is maladaptive. The literature does not yet provide a clear indication which is more likely (Giordano, 2005).

Perhaps the most potent cardioprotection is mediated by signaling cascades activated by IPC, and more recently as described in association with ischemic post-conditioning as well. As reperfusion injury is multi-faceted, it appears that biologic preconditioning is capable of inducing protective responses that also are multi-faceted. Among many mechanisms associated with IPC, activation of the AKT, MAPK, Mek-Erk1/2 pathways, PKCε, HSP 70, mitochondrial K_ATP_ channels, GSK-3β, and HIF-1α regulated genes all have been implicated (Murphy, 2004; Hausenloy et al., 2005; Crisostomo et al., 2006; Zhao and Vinten-Johansen, 2006; Das et al., 2008; Wong et al., 2010). The mitochondria, the source of ATP for the cardiomyocyte, also generate ROS when the tissue is damaged and so much research is aimed at improving mitochondrial energy production and reduce apoptosis to reduce infarct size (Di Lisa et al., 1998; Oldenburg et al., 2002; Krieg et al., 2003; Marin-Garcia and Goldenthal, 2004; Costa et al., 2005; Murphy and Steenbergen, 2007). Inhibiting the phosphorylation of glycogen synthase beta (GSK-3β) has been shown to reduce I/R injury but the mechanism have not yet been fully elucidated (Tong et al., 2002; Gross et al., 2004, 2006, 2007; Murphy and Steenbergen, 2005; Mozaffari and Schaffer, 2008; Omar et al., 2010). Possibilities include modulation of mitochondrial function via the MPTP and the voltage-dependent anion channel (VDAC) but further investigation to illuminate the pathway is needed (Mattson and Kroemer, 2003; Marin-Garcia and Goldenthal, 2004; Di Lisa et al., 2007; Schwertz et al., 2007; Costa and Garlid, 2008; Das et al., 2008; Garlid et al., 2009).

The presumed benefit of independent peripheral oscillators is the ability to anticipate, at the cellular level, changes driven by the SCN in response to environmental cues. Cardiac tissue expresses all known isoforms of *cry* and *per* genes, with *cry2*, *per1*, and *per2* expressed to the greatest degree (Young, 2006). Elements of functioning circadian oscillators are present in cardiac tissue cells:

- Vascular endothelium and smooth muscle show significant circadian variability in functional response (Nonaka et al., 2001; Kawano and Ogawa, 2005; Tsujino et al., 2005; Walters et al., 2006; Viswambharan et al., 2007; Yagita et al., 2010) and dysfunction of these cells types are characteristics of I/R injury (Hazarika et al., 2004; Cozzi et al., 2006).
- Fibroblasts, cells intimately involved in remodeling post-infarction myocardium (Chintalgattu and Katwa, 2004; Squires et al., 2005), show robust circadian responses in culture (Chintalgattu and Katwa, 2004; Nagoshi et al., 2004, 2005; Squires et al., 2005).
- Per2 also has been shown recently to modulate cytokine release in some inflammatory cells (Liu et al., 2006), and inflammation responses are common feature of I/R injury (Welbourn et al., 1991; Jordan et al., 1999; Albert, 2000; Frangogiannis et al., 2002; Albert et al., 2003; Hazarika et al., 2004; Cozzi et al., 2006; Liu et al., 2006).
- Circadian genes appear to regulate a panel of genes encoding for cardiac metabolic enzymes, and it has been postulated that a major role for circadian genes in heart is to synchronize cardiomyocyte metabolic activity (fatty acid vs. glycolytic preferences) with dietary meal induced oscillations in plasma substrate availability. The ability to shift substrates from fatty acid to glycolytic sources is an important characteristic determining functional recovery from I/R injury (Stanley, 2004; Sambandam et al., 2006).

Recent studies examining the role of mPer2 *in vivo* are conflicting. We have shown that mPer2-mutant mice had less severe injury in acute I/R and chronically non-reperfused MI (Virag et al., 2010, 2013). Conversely, Eckle's group has shown that Per2 is cardioprotective and this tolerance may be by virtue of adenosine-elicited stabilization of Per2, modulation of metabolism, and/or inflammation (Eckle et al., 2012; Bonney et al., 2013a,b; Eltzschig et al., 2013). Lipkova et al. showed that Per3 VNTR polymorphism in humans influences the onset of pain associated with acute MI (Lipkova et al., 2014). Alibhai et al. recently demonstrated that post-MI remodeling is worsened by circadian rhythm disruption (Alibhai et al., 2014).

There is clearly a strong confluence between cascades participating in I/R injury and those regulated by circadian genes. Specific and comprehensive studies examining the effects of each circadian gene and/or circadian rhythm variations on myocardial I/R injury, its modulation by IPC, and how these acute responses might influence the subsequent post-infarction remodeling are only recently beginning to be addressed.

## Chronic myocardial infarct repair and circadian rhythm genes

Myocardial infarction is an inflammatory disease. Irreversible ischemic injury begins within 20 min of severe ischemia *in vivo* and is complete within an hour (Holmes et al., 2005). Myocyte, fibroblast, vascular smooth muscle, and endothelial cell death progresses as a wavefront from subendocardial to subepicardial wall (Reimer and Jennings, 1979). Within 24 h, neutrophils infiltrate from the periphery and macrophages follow within 48 h. Neutrophils gradually apoptose by 5 days but macrophages persist in the infarct throughout repair. The breakdown of intercellular collagen fibrils begins by post-translational activation of matrixmetalloproteinases (MMPs). The increased ratio of MMPs relative to tissue inhibitors of matrixmetalloproteinases (TIMPs) propagates this degradation that begins within hours and proceeds for 3–4 days (Vanhoutte et al., 2006). As the matrix disintegrates, myocyte slippage occurs (Cleutjens et al., 1995). Myocyte necrosis, evidenced by contraction bands and pyknosis, as well as dead vascular elements are completely phagocytosed by macrophages within 2 weeks (Baroldi, 1988; Virag and Murry, 2003; Holmes et al., 2005). Between 4 and 7 days granulation tissue, comprised of proliferating fibroblasts and endothelial cells, forms by migration of these cells from the border zone into the infarct core (Virag and Murry, 2003). During this process, a variety of factors are evolved from macrophages, including chemokines such as MCP-1, cytokines such as IL-1 and G-CSF, and ROS such as nitric oxide and superoxide anion (Byrne et al., 2003; Kumar and Jugdutt, 2003; Nian et al., 2004; Frangogiannis and Entman, 2005; Frangogiannis, 2006; Maulik, 2006). These molecules can also cause considerable damage to surrounding viable cells and result in further spreading of the infarct. By 2 weeks, the young scar is characterized by the parallel organization of fibroblasts and predominance of Type I fibrillar collagen (Sun et al., 2002; Virag and Murry, 2003). As the matrix becomes increasingly more fibrotic, endothelial cells, fibroblasts and macrophages begin to apoptose, resulting in vascular regression (Frangogiannis et al., 2002; Virag and Murry, 2003). Further, perivascular fibrosis and disarrayed hypertrophy will not be balanced by the coronary blood flow reserve and the consequent hypoperfusion may cause myocardial ischemia. Progressive thinning and stretching of the non-contractile scar tissue continues, resulting in increased chamber dimensions and increased wall stress. Subsequent volume-overload hypertrophy of the surviving myocardium ensues, but this is insufficient to maintain pressure development. Ultimately, this leads to inadequate pumping and overt heart failure (Pfeffer and Braunwald, 1990; Opie et al., 2006). Cardiac hypertrophy and failure also entail dysfunction of mitochondrial energy and substrate metabolism. Impaired mitochondrial function is associated with a decline in high-energy phosphates, reduced oxygen consumption, and decreased expression and activity of complexes I through IV of the respiratory chain. Therefore, the signaling cascades described above that are active during preconditioning should also be investigated as remodeling progresses. Since circadian clocks in peripheral tissues can disturb and be disturbed by genetic and/or lifestyle- related pathologies, modulation of these mediators may be potentially exploited as novel chronotherapeutic targets for treatment and prevention of these conditions.

## Conclusion

A complex sequence of various cellular activities orchestrate myocardial infarct injury and healing. The importance of circadian genes and/or rhythms is also evident, however, the precise mechanisms are only beginning to be addressed. In depth analyses of macrophage, endothelial cell, and fibroblast behavior *in vivo* in both the acute and chronic phases of infarct repair in genetically modified mice (tissue-specific and global) as well as carefully constructed approaches to sort out cause and effect with respect to circadian genes, rhythm variations, and injury will illuminate signaling cascades that may potentially be exploited to yield more efficacious myocardial infarct healing. This information can then be applied to mitigate myocardial infarction in the presence of pathologies and other confounding variables that may be involved in governing disease severity and progression.

### Conflict of interest statement

The authors declare that the research was conducted in the absence of any commercial or financial relationships that could be construed as a potential conflict of interest.
